# Using eye movement desensitisation and reprocessing (EMDR) with autistic individuals: A qualitative interview study with EMDR therapists

**DOI:** 10.1111/papt.12419

**Published:** 2022-08-11

**Authors:** Naomi Fisher, Henna Patel, Caroline van Diest, Debbie Spain

**Affiliations:** ^1^ Independent Practice London UK; ^2^ King's College London London UK; ^3^ Synthesa Therapy Breda The Netherlands

**Keywords:** autism, clinical supervision, eye movement desensitisation and reprocessing (EMDR), mental health, psychological therapy, trauma

## Abstract

**Objectives:**

Eye Movement Desensitisation and Reprocessing (EMDR) is an evidence‐based psychological therapy that targets distress associated with trauma and affective disturbance. Few studies have examined EMDR for autistic individuals who have co‐occurring mental health conditions, but there is preliminary evidence of effectiveness. The current study explored EMDR therapists' experiences of working with autistic individuals, and adaptations incorporated into clinical practice to make this more accessible and effective.

**Design:**

A qualitative interview design was used. Data were thematically analysed.

**Method:**

Twenty‐three UK‐based EMDR therapists attended one‐off semi‐structured qualitative interviews.

**Results:**

Four main themes emerged: (1) the experience of being autistic; (2) factors around accessing EMDR; (3) adapting EMDR; and (4) supervision and support for EMDR therapists. Participants described offering a nuanced and tailored approach; one that retained the integral components of the eight phases of EMDR, while also being flexible and responsive to each client.

**Conclusions:**

Findings reinforce the importance of offering formulation‐based psychological therapy that flexes in an evidence‐informed way, according to the preferences and needs of autistic individuals. Further research should establish factors influencing accessibility and effectiveness of EMDR for autistic individuals, and the impact of autism‐relevant training on the knowledge, skills and confidence of EMDR therapists and clinical supervisors working with this client group.

**Practitioner points**

- EMDR therapy is increasingly being used to treat co‐occurring mental health conditions experienced by autistic individuals.
- EMDR therapists may need to adapt their approach in order to enable autistic clients to access therapy.
- EMDR therapists in this study emphasised understanding the strengths, preferences and needs of each autistic client, and tailoring therapy accordingly.
- A thorough understanding of autism is important for both EMDR therapists and supervisors, and this is identified as a training need.

## INTRODUCTION

Autism spectrum disorder (henceforth, autism) is a common neurodevelopmental condition, characterised by (1) differences (impairments) in social communication; and (2) restricted or repetitive patterns of behaviour, interests or activities (APA, 2013). As autism is a spectrum condition, the clinical presentation and impact of characteristics day‐to‐day vary. Therefore, understanding the nature of each individual's preferences, needs and strengths, is crucial.

Mental health conditions commonly co‐occur with autism, including anxiety and affective disorders, obsessive compulsive and related disorders, post‐traumatic stress disorder (PTSD), psychosis and eating disorders (for review, see Hossain et al., 2020). Up to 45% of autistic individuals, for example, are reported to have experienced PTSD, which is substantially higher than prevalence rates approximated for non‐autistic individuals (e.g. less than 10% of the adult population; Rumball, 2019). Poor mental health can negatively affect outcomes (e.g. quality of life, independence and occupation). This reinforces the need to advance understanding of putative causal mechanisms for co‐occurring mental health symptoms and develop effective interventions to prevent and treat these.

Research focusing on the feasibility and/or effectiveness of evidence‐based psychological therapies—principally, cognitive behaviour therapy (CBT) and mindfulness‐based interventions—for autistic individuals has increased substantially in recent years (Maddox et al., 2021). Methodologies have become more robust; early intervention studies predominantly described case studies/series and latter studies employed randomised controlled trial (RCT) designs. Conditions most commonly treated are anxiety, obsessive compulsive disorder (OCD) and depression (White et al., 2018).

Psychological therapies typically require adaptation to enhance accessibility for autistic individuals (Kerns et al., 2016); for example, to accommodate social communication differences, sensory preferences/sensitivities and executive functioning difficulties. Adaptations can be made to the therapy structure (e.g. shorter/longer sessions, making sessions more/less structured and a longer/shorter preparation phase), the process (e.g. adapting language used, making the process more transparent and using more/less visual imagery) and content. Ideally, adaptations are identified collaboratively with clients, establishing what works for whom (Kerns et al., 2016; Spain & Happé, 2020; Stark et al., 2021).

Despite the increasing evidence base about psychological therapies for autistic individuals, gaps remain between research and clinical practice (Maddox et al., 2021). Autistic individuals and their families consistently describe systemic barriers to accessing mental health support, including psychological therapy. Barriers relate to service provision (e.g. triage systems poorly aligned to social communication differences and capping of sessions irrespective of need), practitioners (e.g. limited knowledge about autism and uncertainty about how to adapt therapy) and characteristics of autism that may make accessing therapy more challenging (e.g. social communication differences and sensory overwhelm; Adams & Young, 2021; Maddox et al., 2019, 2020).

More research is needed into how evidence‐based therapies can be adapted for autistic individuals, particularly given anecdotal reports that psychological therapists in mainstream mental health services are increasingly asked to work with autistic clients and sometimes lack confidence to do so. Investigating the adaptations that potentially make different types of psychological therapy accessible is, therefore, an important endeavour. The present study focused on one such therapy; Eye Movement Desensitisation and Reprocessing (EMDR) therapy.

EMDR was originally developed as a post‐traumatic stress disorder (PTSD)‐specific therapy (Shapiro, 1989), yet recent studies indicate this is also effective for other conditions, including anxiety and affective disorders, substance misuse and unexplained symptoms (for review, see Sepehry et al., 2021; Valiente‐Gómez et al., 2017, van Rood & de Roos, 2009). EMDR is informed by the Adaptive Information Processing model (Shapiro, 2018). This hypothesises that symptoms in the present day (e.g. flashbacks and somatic re‐experiencing) are due to trauma memories remaining ‘unprocessed’; by processing these memories, we can resolve present day difficulties. A unique aspect of EMDR is that it pairs emotional activation of a memory with bilateral stimulation (BLS), such as through eye movements or other means (e.g. tapping, buzzers and auditory stimuli). It is an 8‐phase protocol, starting with history taking and case conceptualisation, followed by a period of enhancing capacity for using soothing strategies, assessment and elucidation of targets for processing, active desensitisation and trauma processing, and ending with closure and re‐evaluation.

EMDR may be particularly helpful for autistic individuals for several reasons (Fisher & van Diest, 2022). Unlike other psychological therapies, it relies less on spoken communication and assumptions of what is ‘dysfunctional’ or not. It can be accessed by individuals whose language is limited and/or who have social communication difficulties. EMDR adapts well to different ability levels. The standard protocol provides a containing, predictable structure, but within that, EMDR is client‐led (Shapiro, 2018). It does not involve homework or trauma reliving between sessions; something autistic individuals may find overwhelming.

Few studies have focused on EMDR for autistic individuals. These comprise one case study (Kosatka & Ona, 2014), three case series with up to six participants (Barol & Seubert, 2010; Mevissen et al., 2011, 2012) and one RCT with 21 participants (Lobregt‐van Buuren et al., 2019). Each study examined feasibility and/or effectiveness of EMDR for autistic individuals, some of whom had an intellectual disability. Studies incorporated adaptations, including simplifying demands of the protocol or using emotional reactions in the here and now as the target. Overall, findings suggest EMDR is feasible and potentially effective, based on self‐report and Subjective Units of Distress (SUDS) or Validity of Cognition (VoC) ratings.

Adopting a different approach, one recent Delphi survey study garnered 103 EMDR therapists' perspectives about barriers impacting autistic individuals having EMDR (Fisher et al., 2022). The study generated consensus about adaptations EMDR therapists incorporate into clinical practice to make this more accessible and effective (Fisher et al., 2022). Findings highlighted four main barriers to EMDR for autistic individuals, specifically, practitioner‐related characteristics (e.g. poor confidence to work with autistic individuals and uncertainty about how to remain focused), client‐related characteristics (e.g. social communication differences and emotion dysregulation), differences in the therapeutic relationship (e.g. therapist uncertainty about whether an adequate therapeutic alliance had been formed in order to proceed to trauma processing) and systemic issues (e.g. poor understanding of autism or trauma by others in a client's network).

To summarise, we know autistic individuals are at high risk of experiencing co‐occurring mental health conditions, and there is increasing evidence they can benefit from psychological therapies. EMDR is an evidence‐based psychological therapy that has demonstrable effectiveness as a treatment for distress arising from traumatic events and wider mental health conditions, in the general population. There is tentative evidence of effectiveness of EMDR for autistic individuals. We know very little, however, about practitioners' experiences of providing EMDR for autistic individuals. This is important as there may be insights that could benefit from being shared between EMDR therapists to improve clients' experiences and therapeutic outcomes, and to inform future research.

In this study, we explored EMDR therapists' experiences of using EMDR with autistic individuals across the lifespan, as well as adaptations they incorporated into routine clinical practice to make therapy more accessible and effective.

## METHODS

The study methods and findings are reported based on the Consolidated Criteria for Reporting Qualitative Research guidelines (Tong et al., 2007; COREQ; 23, see Supplementary File).

### Research team

The research team comprised four female researchers: three clinical researchers with expertise in working with autistic individuals (one clinical psychologist, and one postgraduate nurse and one postdoctoral nurse and both with post‐qualification training in therapeutic models) and one postgraduate student (HP) with an interest in autism. NF and CvD have substantial EMDR experience. NF and DS have had significant involvement in conducting research, including using qualitative methods.

### Study design

The present study was informed by phenomenological principles. Participants attended for one‐off individual semi‐structured qualitative interviews, conducted via MS Teams (e.g. from work or home), between May and August 2021.

### Ethics approvals

Ethics approvals were obtained REC reference KCL MRA‐20/21‐22833. The participant information sheet outlined reasons for the study and information about researchers' credentials. All participants gave written informed consent.

### Participants

Participants were recruited via inter/national clinical academic networks, special interest groups, EMDR associations and social media, using convenience and snowballing methods. Study inclusion criteria were as follows: (1) inter/nationally based psychological therapists working in any setting, with any clinical population; (2) who had completed at least part of the basic EMDR training (i.e. they had sufficient training to use EMDR with clients); (3) with limited through to advanced experience of using EMDR; and (4) working occasionally, sometimes or often, with autistic individuals.

Twenty‐five EMDR therapists consented to participate. One dropped out before the interview. Responses on the participant questionnaire indicated a second was not eligible. The final sample comprised 23 EMDR therapists. We gathered information about participants' core qualifications, work setting and training in and experience of using EMDR, with a short questionnaire (see Supporting Information for the questionnaire, and Tables 1 and 2).

**TABLE 1:** Participant professional demographic characteristics

	Whole sample (*N* = 23)
Profession^a^	
Clinical psychologist	10
Psychotherapist	7
Cognitive behaviour therapist	5
Mental health nurse	2
Counselling psychologist	2
Psychiatrist	1
Counsellor	1
EMDR training^a^	
Not yet finished basic training	1
Completed basic training	11
Child training level 1	4
Child training level 2	1
Accredited EMDR practitioner	5
Accredited EMDR C&A practitioner	2
Accredited EMDR consultant	6
Training facilitator	1
EMDR experience	
Up to 1 year	3
1–4 years	7
5–9 years	7
10–14 years	4
15 + years	2
Work Setting^a^	
Independent practice	15
Community Mental Health Team	5
Improving Access to Psychological Therapies	4
Tertiary service	4
Education	3
Autism service	2
Inpatient setting	2
Voluntary sector	2
Physical care	1
Social care	1
Forensic service	1
MoD	1
Age of Client Group	
Children and adolescents (<18)	–
Adult (18+)	13
Lifespan	10

^a^ Participants could endorse more than one option.

**TABLE 2:** Participant relevant work experience

	Whole sample (*N* = 23)
Frequency of work with autistic clients	
All the time	4
Regularly (at least a weekly basis)	11
Sometimes (at least once a month)	2
Occasionally (every couple of months or less)	6
Approximately percentage of autistic clients^a^	
None	3
Up to 24%	11
25%–49%	4
50%–74%	2
75%–100%	2
Self‐rated understanding of autism	
5 very well informed. I have attended training and have extensive experience	12
4 quite informed. I have attended some training and have relevant experience	9
3 I have some knowledge of autism	2
2 I have a bit of knowledge of autism	–
1 I know very little about autism	–
Current work with people with intellectual disabilities	
People with severe intellectual disabilities	1
People with mild to moderate intellectual disabilities	6
Not currently working with people with intellectual disabilities	17

^a^ Questionnaire responses were missing for one participant.

### Development of semi‐structured interview schedule and community involvement

We developed the interview based on conversations with autistic adults, health professionals and researchers about the experience of having or delivering EMDR, and evidence‐based psychological therapies for autistic individuals more generally. We sought informal feedback from one autistic adult and a family carer about the interview, which was piloted with a parent/carer (see Supporting Information for the interview). One participant disclosed an autism diagnosis at interview.

### Procedure

Participants completed a short demographic questionnaire. HP and DS conducted the interviews (mean interview duration 39.21 min, range 21.40 to 80.08 min). Participants were not interviewed by researchers they had a working relationship with. Hand notes were taken during the interviews. Transcripts were produced and read alongside the audio‐recording to confirm accuracy. We did not send transcripts to participants for comments or checking.

### Data analysis

Participants' demographic information was summarised descriptively (see Tables 1 and 2). Interviews were analysed thematically (Braun & Clarke, 2006), which involved (1) becoming familiar with the data; (2) generating codes; (3) looking for themes; (4) reviewing tentative themes; (5) assigning labels to themes; and (6) producing a summary of the data.

Transcripts were organised into one master document. DS reviewed all transcripts; 50% of transcripts (*n* = 12) were also independently reviewed by HP. Analysis of the first 12 transcripts started to indicate broad commonalities in participants' responses. We continued with a further 11 interviews (i.e. to a total of 23 interviews), at which time no new themes were identified (see below), and so recruitment ceased at this point.

Analysis involved reading transcripts consecutively and coding data line by line. Codes were categorised and assigned to tentative themes and sub‐themes. Transcripts were then reviewed once more by DS, to see whether there were any additional codes not already encapsulated. Additionally, 10% of transcripts (*n* = 3) chosen at random were reviewed by all four researchers, to compare codes and themes; points identified within these transcripts were comparable for all researchers.

### Reflexivity

We embedded reflexivity throughout the research process. As a research team, we reflected at the outset on our perspectives of autism, neurodiversity and mental health. We also considered ways in which our experiences of working in different health and social care contexts, including in NHS services and independent practice, and providing psychological therapies to autistic and non‐autistic individuals, may have influenced our interest in conducting the study, as well as opinions about focal points for the topic guide. Knowledge of EMDR varied between members of the team, from limited through to advanced (trainer‐level knowledge).

## RESULTS

### Clinical rationale for using EMDR

Participants used EMDR to treat several presenting difficulties, including PTSD, traumatic bereavement, adjustment to adverse life events, phobias, anxiety disorders (e.g. social anxiety and general anxiety), OCD, depression/low mood and addictions.

### Overview of themes

**FIGURE 1:**
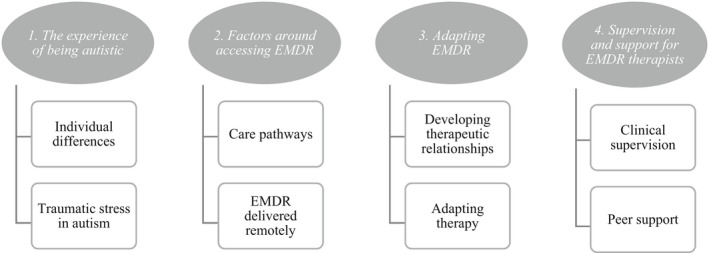
Overview of themes and sub‐themes

There were four main themes of relevance to this paper: (1) the experience of being autistic; (2) factors around accessing EMDR; (3) adapting EMDR; and (4) supervision and support for EMDR therapists (see Figure 1). These were overarching themes, within which sub‐themes were identified, as noted below.

#### Theme 1: The experience of being autistic

The first theme pertained to the experience of being autistic, with two sub‐themes: (1) individual differences and (2) traumatic stress in autism.

##### Individual differences

Many participants perceived that autistic individuals present with individual differences. They felt EMDR therapists should assess and accommodate the uniqueness each client presents with, rather than tying the therapeutic approach/intervention to a diagnosis per se.

… all of us, whether we're in inverted commas, neurotypical or not, have elements of us which are different in different ways. So everybody has sensory differences. Everybody has different ways of recalling information. Everybody has different levels of emotional literacy and interoception. So I treat everybody as an individual … everybody gets [a] bespoke level of EMDR.

Participants highlighted clients may be undiagnosed or misdiagnosed; again, reinforcing the importance of not making assumptions.

[I have learnt] to be very sceptical about diagnoses … a person I worked with recently came with an ADHD [diagnosis] but actually conceptually, she had ASD.

##### Traumatic stress in autism

Many participants talked about traumatic stress experienced by autistic individuals, in particular, contributory factors and diagnostic overshadowing.

###### Contributory factors

Summarising consensus, one participant identified, ‘lots of things can kind of start to cluster together’, including ‘big traumas and little [t] traumas’.^1^ Most said ‘lower level incidents’ might not seem traumatic to a non‐autistic person, yet are ‘really significant’ for some autistic individuals. Adverse life events, including victimisation and ‘social and asocial misunderstanding’, were frequently reported. Other factors included ‘sensory overwhelm’ and ‘unfamiliar situations’. Some participants described potential trauma resulting from ‘being in the world where maybe they [individuals] often don't know that they're autistic’, and ‘trying to work out how [they fit] into an autistic world or non‐autistic world with an autistic brain’.

The longitudinal nature of undetected trauma exposure was highlighted.

It's sort of an ongoing trauma situation [school], but you're really trying to help stabilise these children, because they are basically all very uncontained and out of the window of tolerance nearly all the time.

Participants also noted ways in which ‘the world isn't set up for autistic people’, and the detrimental impact this can have.

There's almost an implicit sense that, well, you [autistic individuals] just have to fit in, whereas we would probably never make that statement about any other sort of diversity characteristic; we'd expect the world to be flexible.

##### Diagnostic overshadowing

Diagnostic overshadowing was mentioned, ‘if you actually look at some of the sort of PTSD responses and autism sort of presentations, they actually overlap’; for example, dissociation, characteristic of PTSD versus shutdowns seen in autism. Participants emphasised not ‘everything fits nicely into one or the other box [autism and PTSD]’, Therefore, individualised formulation, outlining symptom trajectory and modifiers, seems key.

#### Theme 2: Factors around accessing EMDR

The second theme pertained to service provision, with two sub‐themes: (1) care pathways and (2) EMDR delivered remotely.

##### Care pathways

Several participants noted routes to EMDR lack clarity and may not be considered by gatekeepers.

What are the pathways for somebody to actually get EMDR? What assumptions are being made about people as to whether they could respond to EMDR?

NHS employees reported pressure to ‘get half of them [clients] better … we've got a short timeframe. We've got to get through as much as we can’. Acknowledging some autistic individuals benefit from a longer course of therapy, some participants used initial therapy sessions to ‘get a better understanding of the person's unique experience of their autism’, or ‘spend the first block understanding the autism and stabilisation, and then have a break [a pause in therapy] and come back [to trauma‐focused approaches]’.

Participants talked about why they would consider using EMDR as opposed to another trauma‐focused evidence‐based therapy (e.g. cognitive or CBT). Many described that the structured format of EMDR and limited emphasis on ‘having to talk about thoughts and feelings’ resonated with autistic clients, especially if presenting with trauma or ‘to help somebody resolve something they've been carrying around for a long time’.

It's not a talking therapy. It's not reliant on the communication and the miscommunication that can creep in, and having to sit opposite and having to make eye contact and things like that. I mean, they do keep their eyes open but they're not looking.

Participants frequently commented on the significance of clients being able to process trauma in their own way, and the intrinsic validation provides at a time autistic individuals are likely to feel vulnerable.

[Clients can] move the processing in a way that makes specific sense to them … they can do it in a way that works for them, and they don't have to have people going that's not quite how I see it. You can support them to make sense of their world with their processing.

I've talked a lot about the structure [of EMDR] and that being quite containing, but sometimes I wonder if the opposite is also true; that we say you don't have to try to control what comes up when it's happening [actively processing trauma] … I'm not having any expectations of you; I'm not going to ask you to do any mental gymnastics or anything like that; just see what happens and that freedom from the expectations and from the ‘I'm supposed to do something here. I'm supposed to talk about. My problems are…’ The freedom from that can allow people to let go a little bit and maybe not worry about that aspect in the session.

One participant said they ‘explain all the different options that we have [i.e. therapeutic modalities] and then which ones I think might be more helpful’, to empower autistic individuals to make evidence‐informed choices.

##### EMDR delivered remotely

Prior to the pandemic, few participants did EMDR online, with several remarking ‘it was the most ridiculous notion’. However, pandemic‐related restrictions forced online ways of working. Many said EMDR delivered online is far more ‘effective’ than anticipated, and a preferred option for some autistic individuals.

I think when you've got a patient who's coming into your space [the therapist's office] and you're very much dictating the space, and it's unfamiliar, and they've got to totally adapt to where you are … My guess is that for the vast majority of people on the spectrum, working online would be their preference.

Adaptations to online working were described; for example, formally agreeing practicalities such as where the client would sit, head height and proximity to the camera, having a back‐up plan if the internet connection failed and developing a crisis/safety plan if clients dissociated. Participants were creative about the forms of BLS used (e.g. ‘drumming with cake tins’) and used adjustable BLS via video platforms.

Several participants described worry about how clients might manage their distress after sessions had ended (i.e. that they could not metaphorically ‘leave their problems behind’ in the therapist's office). Conversely, participants described that being in a familiar environment may in fact be conducive to clients processing trauma.

Some participants used a mixed model.

Mostly I prefer to meet people first and then do a hybrid thing of like when the weather is bad, we'll work online and when the weather is good, we'll meet in person.

#### Theme 3: Adapting EMDR

The third theme focused on using EMDR with autistic individuals, with two sub‐themes: (1) developing therapeutic relationships and (2) adapting therapy.

##### Developing therapeutic relationships

Participants felt that having an understanding of autism helped them develop therapeutic alliances with clients.

I think understanding autism really helps to develop a therapeutic relationship, because they've [clients] spent their whole lives feeling, you know, that other people don't understand them.

Them [clients] feeling you know that you can support them to change in the ways that they need to, but you're not going to ask them to do things they can't do.

Participants prioritised being open and curious with clients, including about autism and what this means to or for them.

Be warm and friendly and open. Just let that person know that you are listening to them and that you care about them and that whilst you might not be autistic yourself, that that's not a barrier at all and you want to learn from them as well.

Awareness of social communication differences was deemed necessary. Consequently, they might be more ‘concrete in my use of language’, and explicitly observe cues.

Some people with autism maybe don't have this same emotional expression, so they might not be nodding along when you're speaking or smiling when you smile at them. It doesn't mean that they're not forming a good relationship with you.

It's being alert to signals … to be on the lookout for what might be telling me how this person likes to interact.

##### Adapting therapy

Participants described providing EMDR to autistic clients.

###### General factors

Participants emphasised the standard EMDR protocol provides a containing structure some autistic clients like, while also enabling them to remain flexible and client‐centred. Participants worked flexibly, depending on how the client managed at different points.

Their emotional literacy and their ability to socially communicate will vary depending on their stress level. So if you're dealing with a high SUDS incident, their ability to give negative and positive cognitions or a VOC [rating scale] would be significantly impaired until you've done some of the work ….

###### History taking and case conceptualisation

Some therapists talked about using this phase to distinguish between trauma and autism.

The assessment phase is really picking apart what can be understood as part of the trauma presentation and what might actually be autism … so, for example, repetitive play, you know, is the child playing the same thing over and over again? Because it's comforting. It's familiar, it's predictable, or only them trying to process their trauma through play?

While some participants conducted a neurodevelopmental assessment, others focused on discussing autism, neurodiversity, and what this means to each client.

The first thing that I would do would be very much open up the conversation about their neurodivergence and neurodiversity … we're very much opening up a conversation about different brains and about how come different people's minds work differently.

Others noted the importance of specific assessments potentially relating to a client's ability to do EMDR.

So, for example, I might want to know, do they visualize or not? You know, are they able to do images and things because there's an awful lot of assumptions in EMDR that people can visualize, can create an image, can use that as their resourcing. Do they have other neurodivergent issues? Are they able to kind of concentrate? What sort of cognitive load do they already have in if they're trying to deal with face to face interactions or Zoom interactions? If they're trying to interpret spoken language, it's about opening up assumptions rather than ruling them out.

###### Preparation

Participants took time to explain clearly what would happen in therapy.

Some people really want to know the ins and outs in great detail of the AIP model the whole way the brain works. I only have a scant knowledge, and I'll say, “well, this is what I know in relation to EMDR. These are some places that you might go and actually look up some more information about trauma or your nervous system.” I always make sure that there's ways to access the information if they can't access it with me.

Participants emphasised flexibility and responsiveness. Some used a longer preparation phase. Some noted how imaginative clients could be.

I've had some of the autistic kids come up with the most wonderful containers, and I mean I saw this one boy who put his stuff in a shipping container that was sent to the Antarctic and was hidden in a cave guarded by Penguins with lasers.

Others talked about difficulties using imaginative techniques.

[I] tried to do some guided fantasy relaxation work … he doesn't do imagination at all … he doesn't do “imagine you are anywhere” … “what do you mean?” … “Imagine I'm here?”

Others described unexpected interpretations of standard exercises.

Mindfulness “leaves on a stream” exercise; she did not take it the way that I thought that she was. I found out later that she was actually sending her thoughts over a cliff edge and destroying them, which isn't the point of mindfulness but never mind she made it work for her.

Several participants used concrete examples for the safe place exercise.

Rather than kind of imagining a new novel place which she really struggled with, she was able to say, well, I've been to this this place before, so I'll just remember going there. And it was really a lovely place.

###### Assessment phase (the ‘set‐up’ phase for trauma processing)

Participants reported flexibility when identifying a target image and negative and positive cognitions; a key part of the set‐up phase for EMDR processing.

I think even for [clients] that don't require a lot of adaptation though, the one thing I have to be a bit flexible around is getting that worst image in phase three. It may be that an image or the visual quality may not be very strong. You might be working more with a feeling in the body … I make a point of saying “it might be a picture you see, or a sound or a feeling in your body”.

I had other clients who can get the negative cognition but can't get the positive or can get everything, but you spend so much time trying to tie down the negative cognition that in the end you go with a general feel of it.

Others found some cognitions reoccurred and it was difficult to establish these were interpretations rather than fact.

Cognitions were a bit hard to pin down because she takes it quite literally … “obviously that I am no good” she said.

Participants mentioned some difficulties in locating body sensations. They had learnt to go with whatever the client brought.

She doesn't feel totally in her body … [she says] my legs do feel a bit heavy, and we just go with that rather than getting her to really explain it.

###### Trauma processing

There was no consensus about optimum forms of BLS; this was client‐specific. Some participants used more than one form of BLS concurrently.

It's not so much one will work better [type of BLS], but you need more than one at the same time.

Running on the spot or stamping or butterfly hugs.

I have tended to use eye movement and change direction if I felt something was kind of blocked and all of the clients that I have seen have been able to tolerate that

Initially I used a wand in the office and she that didn't like that. That didn't seem to fit right with her sensory profile and what we finally found worked was using the cloud EMDR app, but we had to spend some time tinkering around the speed.

Others talked about accommodating clients' sensory sensitivities.

The noise of the tapping on her jumper [butterfly taps] and the noise that the fabric was making, it was just really distracting and irritating to her … so we switched to e‐taps.

We did an experiment with buzzers, and she didn't like those because there was too much physical touch

Several participants said they found it more difficult to interpret facial expressions during processing.

When I'm working with … neurotypical people, I watch their faces and I get a bit more … I don't get those clues [with autistic people] … they don't seem to show me what's going on inside as much.

I'm used to working with a client group that has lower nonverbal communication and therefore being particularly [aware of] the breathing. The deep breath that happens after something has shifted or tensing and holding the upper body, all of those very subtle things.

Our last session was really heavy, and I hadn't actually picked up that it was heavier than any of the other ones because I wasn't picking it up from her.

I think that's possibly why I like the sitting close because then you really can pick up on the really tiny ones [cues]

Some commented clients could experience difficulty explaining their feelings, so they emphasised this is not essential.

Something that might come up more often in autism is clients who find it difficult to verbalize their inner experience. When you're stopping and saying “okay, what did you get?”, they might not be able to verbally articulate that. It might be worth having a bit of a discussion with the person about that; that EMDR will still work even if you're struggling to put it into words.

#### Theme 4: Supervision and support for EMDR therapists

The fourth theme concerned support for participants, with two sub‐themes; (1) clinical supervision and (2) peer support.

##### Clinical supervision

This was identified as integral to clinical practice. Some participants had two supervisors; for example, one providing input for child and adolescent work, and a second for work with adults. A lack of autism knowledge and expertise in practitioners and/or supervisors was commonly reported, and potentially problematic, as this could adversely impact formulation and safety of practice.

The problem, I think, would be if you've got somebody who's working with a client who has got autism and doesn't know much about autism, and their supervisor doesn't know much about autism either.

Several participants suggested autism training should be built into the EMDR accreditation process.

As an EMDR supervisor you could potentially have a lot of supervisees, and if each of those supervisees has two or three autistic people on their caseload, then there's the potential for that knowledge via the consultant to reach a lot of patients.

##### Peer support

The final sub‐theme concerned peer support. Participants described feeling a sense of containment from the EMDR community.

There's resources coming out of your ears … I'm sort of joining an establishment, and I feel supported to be able to do it [EMDR]

Another said the pandemic had opened up possibilities for ‘a whole range of training with different facilitators across the world’.

## DISCUSSION

To our knowledge, this is the first qualitative study focusing on EMDR therapists' experiences of working with autistic individuals. Participants were enthusiastic about using EMDR and described many practical ideas about adapting therapy, while retaining key elements of the standard protocol. Participants' EMDR experience varied, but in order to train in EMDR, a person must be a qualified mental health professional, so all had prior professional experience. Twelve participants rated themselves as being ‘very well informed’ about autism and a further nine said they were ‘quite well informed’. EMDR experience was more variable; 10 participants had up to 4 years of experience, and only six reported 10+ years of experience.

### Individual differences

The overriding theme emerging from the data was the need to understand and appreciate each client's unique profiles, and how this might affect their ability to access EMDR. Participants considered numerous factors, based on their understanding of autism; for example, whether a client would be able to visualise and use imagery, the sensory input in a room and what forms of BLS would be most appropriate.

Participants talked about abandoning preconceptions about autism, and understanding the client in front of them. Some of their responses challenge stereotypes often held about autistic individuals. Some clients were very imaginative, while others struggled to imagine scenarios. Some clients disliked buzzers, while others really liked them. Participants described needing to be particularly sensitive to the potential for clients experiencing overwhelm, as some may not express their emotions openly. Challenging stereotypes of autism seems especially pertinent, as prior research has found that professionals' biases about autism/autistic individuals, can adversely impact care (Como et al., 2020; Fennell & Johnson, 2021). Moreover, therapists' attitudes can predict intentions and motivation to provide psychological therapy (CBT) for autistic individuals, with more favourable attitudes linking to greater intentions (Maddox et al., 2019).

Study findings highlighted the diverse experiences autistic clients bring to therapy, in particular the prevalence of intense and persistent reactions to a range of what might be considered ‘lower level traumas’ or ‘little t trauma'. This is consistent with findings from qualitative (Kerns et al, in press) and quantitative (Rumball et al., 2020) research, indicating some autistic individuals experience trauma responses to subjectively aversive events. Previous studies also report that practitioners may not adequately screen/assess for trauma in autistic individuals (Kerns et al., 2020), and diagnostic overshadowing—highlighted by participants here and elsewhere (e.g. Kildahl et al., 2020)—further complicates assessment and formulation. Taken together, more research is needed to better understand the range and impact of trauma experienced by autistic individuals, to inform refinement of autism‐adapted and validated screening/assessment tools.

Crucially, the AIP model makes no assumptions about the nature of traumatic events; this focuses on the psychological impact on the individual, rather than the nature of the event itself (Shapiro, 2018). Participants used EMDR for numerous subjectively aversive events. This is quite different to the way in which PTSD is defined, which requires exposure to a ‘Criterion A' event, involving actual or threatened death, serious injury or sexual violence (APA, 2013). Many NHS services in the UK offer EMDR only to individuals meeting formal PTSD diagnostic criteria. This means autistic individuals could be effectively barred from receiving a potentially effective evidence‐based approach, compounding existing barriers to psychological therapy. Further consideration is needed regarding whether PTSD diagnostic criteria are sufficiently sensitive to detect traumatic stress in autistic individuals (Brewin et al., 2019).

### Service provision

Participants working in the NHS reported barriers to providing EMDR to autistic individuals due to unclear service pathways and service restrictions. They described pressure to demonstrate clients' improvement rapidly, making it difficult to offer extra time autistic clients might need. A systematic review of barriers and facilitators for psychological therapy for autistic individuals identified inadequate service provision as a recurrent theme (Adams & Young, 2021). Potentially, inflexible service models have a particular impact on autistic individuals, given that research shows how important flexibility is with this client group. Moreover, it is possible that a poor experience of service provision deters autistic individuals from help‐seeking at future points. Given high rates of co‐occurring conditions, studies focusing on strengthening facilitators and mitigating barriers to health care for autistic individuals are crucial.

### Adapting EMDR for autistic individuals

Participants emphasised flexibility, responsiveness and being ready for the unexpected. All sometimes adapted the structure, process or content of EMDR. This is in keeping with the general literature about psychological therapies for autistic individuals (Kerns et al., 2016; Spain & Happé, 2020; Stark et al., 2021), as well as EMDR intervention studies (Barol & Seubert, 2010; Kosatka & Ona, 2014; Lobregt‐van Buuren et al., 2019; Mevissen et al., 2011, 2012), a recent Delphi survey study about barriers and adaptations to EMDR (Fisher et al., 2022) and two unpublished EMDR autism guidelines (Lievegoed et al., 2013; Sherri Paulson, personal communication).

It is possible that therapists may benefit from a checklist/toolbox of questions to ask themselves, or possibilities for adaptations, rather than a list of assumptions about the way in which autistic people will respond to EMDR. Rather than assuming, for example, that autistic people will dislike eye movements, it may be useful to consider their experience of different forms of BLS. Rather than assuming they will have difficulties with visual imagery, it could be helpful to discuss whether imagery is something that comes more/less easily to them. In this way, therapists can hold in mind difference, while also making few assumptions about clients. Further studies could clarify whether development of a checklist—co‐produced with stakeholders—results in more accessible and effective therapy.

### Supervision and support for EMDR therapists

Participants consistently mentioned the importance of autism‐relevant training for practitioners and clinical supervisors. Prior research shows that practitioners (Corden et al., 2021) and psychological therapists (Lipinski et al., 2022) working in non‐autism services can lack knowledge, skills and confidence to work with autistic individuals. Few, if any, clinical training courses include substantial focus on autism. Improving autism awareness and skills in core and post‐qualification training for therapists (e.g. EMDR training) could result in a better tailored care pathway for autistic individuals. Additionally, autism‐relevant training is not currently part of the EMDR supervisors' training; therapists can be supervisors with limited autism knowledge. Given increased rates of autism diagnosis (Russell et al., 2022), incorporating relevant training should be considered a matter of urgency. The degree to which therapist knowledge and understanding autism influences outcomes requires further research.

### Limitations

We could not ascertain how many EMDR therapists saw the study flyer but opted not to participate. They may, conceivably, have different views or experiences to participants. We included EMDR therapists with wide‐ranging experience, but did not independently measure autism knowledge, past relevant training or expertise. As is common, the topic guide included some questions that could be construed as leading; more open questions may have gleaned additional comments. We ceased interviewing as data analysis indicated commonalities in codes and categories across transcripts. Participants were not asked whether there were other people in the vicinity during study participation (e.g. that could have influenced content of conversation), but there were no obvious interruptions interviewers were aware of. We did not send transcripts to participants for comments or checking. We did not, purposively recruit participants so that there were sub‐groups of EMDR therapists working with young people and adults, individuals with and without concurrent intellectual disability, or across different types of services and settings Findings may therefore not be generalisable to all autistic individuals and EMDR therapists.

### Implications

Study findings reinforce the importance of offering formulation‐based EMDR therapy that flexes in an evidence‐informed way, according to the preferences and needs of autistic individuals. Some autistic individuals are likely to benefit from more time for assessment and setup phases for EMDR. It may be prudent for EMDR therapists to discuss this at the outset with supervisors or service managers, in order that autistic clients are able to access the number of sessions likely needed, rather than a prescribed amount. Exploring autistic clients' preferences, such as for grounding exercises and forms of BLS, is key to accessibility and effectiveness of EMDR. Some autistic clients may benefit from being shown different examples of BLS and testing these out. Finally, we advocate that accessing adequate autism‐informed clinical supervision is crucial for ensuring safety of practice.

## CONCLUSION

EMDR may be an effective psychological therapy for autistic individuals who experience co‐occurring mental health conditions, but few studies have examined this approach. Here, we interviewed 23 EMDR therapists about their experiences of working with autistic individuals. Collectively, participants described using an iterative, formulation‐based approach; trying things out and being ready to abandon strategies if these did not work. Adaptations to the standard eight‐phase protocol were commonly incorporated, but participants articulated that this should be determined on an individual basis. Further qualitative, quantitative and interventional research examining accessibility and effectiveness of EMDR, from the perspectives of autistic individuals, families and practitioners, is warranted.

## AUTHOR CONTRIBUTIONS

**Naomi Fisher:** Conceptualization; formal analysis; methodology; supervision; writing – original draft. **Henna Patel:** Formal analysis; investigation; methodology; project administration; validation; writing – review and editing. **Caroline van Diest:** Conceptualization; formal analysis; methodology; validation; writing – review and editing. **Debbie Spain:** Conceptualization; data curation; formal analysis; investigation; methodology; project administration; supervision; validation; writing – original draft.

## CONFLICT OF INTEREST

All authors declare no conflict of interest.

## Supporting information


Appendix S1

## Data Availability

The dataset is not available to individuals outside of the research team due to ethical restrictions. Participants consented to anonymised quotes being used for dissemination purposes, but not for the sharing of transcripts.

## Footnotes

^1^
Little ‘t’ traumas are defined as events that would not meet formal diagnostic criteria for PTSD, but that are subjectively traumatic.
