# Supplementary material for: Using eye movement desensitisation and reprocessing (EMDR) with autistic individuals: A qualitative interview study with EMDR therapists
Source: Psychol Psychother. 2022 Aug 11;95(4):1071–89. doi: 10.1111/papt.12419 (PMC9804816; doi:10.1111/papt.12419)
Supplement: Supplementary file 1 — Appendix S1 [file PAPT-95-1071-s001.docx]

**Supplementary material**

**The EPAS 2 participant questionnaire**

Demographic Questions:

1. What is your core clinical profession? Please highlight all that apply.
2. Arts psychotherapist
3. CBT therapist
4. Clinical psychologist
5. Counsellor
6. Counselling psychologist
7. Drama psychotherapist
8. Educational psychologist
9. Forensic psychologist
10. Learning disabilities nurse
11. Medical Dr (e.g. GP)
12. Mental health nurse
13. Mental health social worker
14. Occupational therapist
15. Psychiatrist
16. Psychotherapist (please specify your approach)
17. Other. Please specify
18. What country are you based in?
19. What setting do you work in? Please highlight all that apply.
20. Children and young people’s psychological therapies service/CAMHS
21. Specialist mental health service (tertiary care)
22. Adult psychological therapies service, or community mental health service (secondary care)
23. IAPT (primary care)
24. CYP IAPT
25. Inpatient mental health setting
26. Physical health community service (outpatients clinic)
27. Specialist service for autistic people
28. Specialist service for people with an intellectual disability
29. Private practice
30. School
31. University
32. Forensic services
33. Voluntary Sector organisation.
34. Other (please provide more information)
35. What is the age range of people you work with? Please highlight the relevant options.
36. Children (up to 12)
37. Adolescents (13-19 years old)
38. Working age Adults
39. Older Adults (aged 65 and upwards)
40. Young people and adults
41. What difficulties do you use EMDR for when working with autistic people? Please highlight all that apply.
42. Post-traumatic stress (PTSD)
43. General Anxiety
44. Depression/Low mood
45. Traumatic bereavement
46. Social anxiety
47. Adjusting to adverse life events
48. Obsessive compulsive symptoms
49. Addictions
50. Specific Phobias
51. Challenging behaviour
52. Other (please specify)
53. What level of EMDR training/accreditation have you completed? Please highlight all that apply.
54. Not yet finished basic training
55. Completed Level 3 or 4 (finished basic training)
56. Child Training Level 1
57. Child Training Level 2
58. Accredited EMDR Practitioner
59. Accredited EMDR C&A Practitioner
60. Accredited EMDR Consultant
61. Accredited EMDR C&A Consultant
62. Training Facilitator
63. Trainer
64. For how many years have you provided EMDR as part of your clinical or academic role?
65. How often do you work clinically with autistic people? Please highlight all that apply.
66. All the time (everyone you work with is autistic or has suspected autism)
67. Regularly (at least on a weekly basis)
68. Sometimes (at least once a month)
69. Occasionally (every couple of months or less)
70. Approximately what percentage of the people you currently work with have a confirmed autism diagnosis?
71. Are you working with people who you suspect might have autism or who have significant autistic traits, but who do not have a formal autism/Asperger/ASD diagnosis? What (approximate) percentage of your case load?
72. Yes
73. No

Percentage:

1. Do you work with people with intellectual disabilities?
2. Yes - severe
3. Yes – mild/moderate
4. No
5. Do you work with people with challenging behaviour?
6. Yes – severe
7. Yes – mild/moderate
8. No
9. How would you rate your knowledge and understanding of autism?

5 : very well informed, I have attended training and have extensive experience

4 : quite informed, I have attended some training and have relevant experience

3 : I have some knowledge of autism

2 : I have a bit of knowledge about autism

1 : I know very little about autism

**The EPAS 2 interview schedule**

**Introduction:**

In this interview, I would like to ask about your thoughts about and experiences of using EMDR with autistic people. The interview is split into two main sections:

1. Your thoughts and experiences of using EMDR with autistic people
2. Your thoughts about how training or supervision could be improved so that EMDR practitioners have more knowledge, skill and confidence in working with this clinical group

**Thoughts about and experiences of using EMDR with autistic people:**

- Can you tell me a bit about your experience of using EMDR with autistic people?
  - Do you think using EMDR with an autistic person differs from working with a neurotypical person?
    - How?
    - I am not sure how familiar you are with the phases – and I should say that we are recruiting therapists with a range of experience – but if you use that terminology [i.e. phrases], is there anything else you adapt at any point?
    - Would it be okay to ask for a couple of examples of adaptations?
  - Why would you use EMDR as opposed to another therapeutic approach with an autistic client, for example CBT?
  - What do you really like about using EMDR with autistic people?
  - Are there any parts of EMDR that seem particularly effective with autistic clients? What?
  - Are there particular types of BLS that you think might be more or less accessible for autistic clients?
  - Are there specific BLS that you prefer?
  - Are there any aspects of EMDR that seem less relevant or effective when working with autistic clients?
    - What?
- Is there a difference in the therapeutic relationship you establish with autistic people?
  - Are there things you do differently to facilitate the therapeutic relationship?
- What are the things you would like other practitioners to know about in terms of using EMDR with autistic people?

**Adaptations:**

- Are there any other additional adaptations you incorporate in EMDR for autistic people that you haven’t mentioned? This might be things you do more of, or less of, or differently, to accommodate needs and preferences of autistic people.
- Is there anything that you do differently in terms of closing down a processing session when working with autistic clients?
- [**Supplementary question if the person works with children**: are there any adaptations or theories or models that you blend into EMDR when working with younger children?]
- [**Supplementary question if working with someone with a learning disability**: if you adapt more for a client’s learning disability, what do you do?]

**Outcomes:**

- How do you measure change or outcomes?
- Do you use standardised outcome measures?
  - If so, do you think that these are useful and applicable to autistic clients?

**Working Remotely:**

- Are you using EMDR remotely?
- What do you think about working in this way?
- Are there any extra considerations or adaptations needed to work in this way with autistic people?

**Improving Delivery of EMDR:**

- In what ways do you think we could improve provision of EMDR for autistic people?

**Training and Supervision:**

- Are there any additional considerations in terms of clinical supervision or training for supervisees or supervisors?

**COREQ checklist**

(Tong, A, Sainsbury P, Craig J. Consolidated criteria for reporting qualitative research (COREQ): a 32-item checklist for interviews and focus groups. International Journal for Quality in Health Care 2007; 19(6):349-57. PMID: 17872937).

| **Topic** | **Item number** | **Reported on page no.** |
| --- | --- | --- |
| Domain 1: Research team and reflexivity | | |
| *Personal characteristics* | | |
| Interviewer/facilitator | 1 | Page 11 |
| Credentials | 2 | Page 6 |
| Occupation | 3 | Page 6 |
| Gender | 4 | Page 6 |
| Experience and training | 5 | Page 6, 11 |
| *Relationship with participants* | | |
| Relationship established | 6 | Page 11 |
| Participant knowledge of the interviewer | 7 | Page 7 |
| Interviewer characteristics | 8 | Page 12 |
| Domain 2: Study design | | |
| *Theoretical framework* | | |
| Methodological orientation and theory | 9 | Page 7 |
| Sampling | 10 | Page 7 |
| Method of approach | 11 | Page 7, 8 |
| Sample size | 12 | Page 8, 32 |
| Non-participation | 13 | Page 8 |
| *Setting* | | |
| Setting of data collection | 14 | Page 7 |
| Presence of non-participants | 15 | Page 7 |
| Description of sample | 16 | Page 9, 10 |
| *Data collection* | | |
| Interview guide | 17 | Page 11 |
| Repeat interviews | 18 | Page 7 |
| Audio/visual recording | 19 | Page 7 |
| Field notes | 20 | Page 11 |
| Duration | 21 | Page 11 |
| Data saturation | 22 | Page 11 |
| Transcripts returned | 23 | Page 11 |
| Domain 3: Analysis and findings | | |
| *Data analysis* | | |
| Number of data coders | 24 | Page 11 |
| Description of the coding tree | 25 | Page 13 |
| Derivation of themes | 26 | Page 11-12 |
| Software | 27 | Page 11 |
| Participant checking | 28 | Page 11 |
| *Reporting* | | |
| Quotations presented | 29 | Page 14-27 |
| Data and findings consistent | 30 | Page 12-27 |
| Clarity of major themes | 31 | Page 14-27 |
| Clarity of minor themes | 32 | Page 14-27 |

**Example of data analysis**

| **Examples of coded data from transcripts** | **Sub-theme** | **Theme** |
| --- | --- | --- |
| Have never considered doing therapy virtually  COVID-19  Didn’t feel terribly confident to switch online  Had far greater success  Pleasantly surprised but it’s been a scary journey  Working online would be their preference  You can only establish the therapeutic relationship when you’re working in the room  The lack of stress from having to travel  The convenience of it  Cheaper option  Their own environment with a lot of familiar things  Practical things with the screen freezing  A good internet connection  The screen cut out, so that’s a bit more distressing  The movement is so narrow on Zoom  Can’t see all the body language  Camera should be tilted down a bit further  I can’t do EMDR with mobile phones  Basic rules of etiquette  Privacy and making sure that people have a safe space  You’re not in the same room, need a back-up plan  Have they got the support they need after the session  Putting your memories in that drawer there and walk away  You might miss a bit of dissociation  Using drumming with cake tins online  Bilateral base  Hybrid thing | EMDR delivered remotely | Factors around accessing EMDR |
